# Stage-Based Mobile Intervention for Substance Use Disorders in Primary Care: Development and Test of Acceptability

**DOI:** 10.2196/medinform.7355

**Published:** 2018-01-02

**Authors:** Deborah Levesque, Cindy Umanzor, Emma de Aguiar

**Affiliations:** ^1^ Pro-Change Behavior Systems, Inc South Kingstown, RI United States

**Keywords:** pilot projects, substance use disorders, primary care, behavioral medicine, expert system

## Abstract

**Background:**

In 2016, 21 million Americans aged 12 years and older needed treatment for a substance use disorder (SUD). However, only 10% to 11% of individuals requiring SUD treatment received it. Given their access to patients, primary care providers are in a unique position to perform universal Screening, Brief Intervention, and Referral to Treatment (SBIRT) to identify individuals at risk, fill gaps in services, and make referrals to specialty treatment when indicated. Major barriers to SBIRT include limited time among providers and low motivation to change among many patients.

**Objective:**

The objective of this study was to develop and test the acceptability of a prototype of a mobile-delivered substance use risk intervention (SURI) for primary care patients and a clinical dashboard for providers that can address major barriers to SBIRT for risky drug use. The SURI delivers screening and feedback on SUD risk via mobile tools to patients at home or in the waiting room; for patients at risk, it also delivers a brief intervention based on the transtheoretical model of behavior change (TTM) to facilitate progress through the stages of change for quitting the most problematic drug and for seeking treatment if indicated. The prototype also delivers 30 days of stage-matched text messages and 4 Web-based activities addressing key topics. For providers, the clinical dashboard summarizes the patient’s SUD risk scores and stage of change data, and provides stage-matched scripts to guide in-person sessions.

**Methods:**

A total of 4 providers from 2 federally qualified health centers (FQHCs) were recruited for the pilot test, and they in turn recruited 5 patients with a known SUD. Furthermore, 3 providers delivered dashboard-guided SBIRT sessions and completed a brief acceptability survey. A total of 4 patients completed a Web-based SURI session and in-person SBIRT session, accessed other program components, and completed 3 acceptability surveys over 30 days. Questions in the surveys were adapted from the National Cancer Institute’s Education Materials Review Form. Response options ranged from 1=strongly disagree to 5=strongly agree. The criterion for establishing acceptability was an overall rating of 4.0 or higher across items.

**Results:**

For providers, the overall mean acceptability rating was 4.4 (standard deviation [SD] 0.4). Notably, all providers gave a rating of 5.0 for the item, “The program can give me helpful information about my patient.” For patients, the overall mean acceptability rating was 4.5 (SD 0.3) for the mobile- and provider-delivered SBIRT sessions and 4.0 (SD 0.4) for the text messages and Web-based activities. One highly rated item was “The program could help me make some positive changes” (4.5).

**Conclusions:**

The SURI program and clinical dashboard, developed to reduce barriers to SBIRT in primary care, were well received by providers and patients.

## Introduction

### Substance Use Disorders in Primary Care

Data from the Substance Abuse and Mental Health Services Administration’s 2016 National Survey on Drug Use and Health indicate that 21.0 million Americans aged 12 years and older (8.1%) needed treatment for a substance use disorder (SUD) in the past year [1]. The annual economic costs associated with SUD are estimated at US $193 billion for illicit substance use [2], US $78.5 billion for prescription opioid misuse [3], and US $249 billion for excessive alcohol use [4] because of lost productivity, health care costs, and criminal justice costs. SUD is under-recognized and under-treated; in 2016, only 10.6% of individuals requiring treatment for an SUD received it [1]. Although only a minority of individuals with an addiction seek specialty treatment [5], an estimated two-thirds see a primary care or urgent care provider every 6 months [6]. Given their access to patients, primary care providers are in a unique position to perform Screening, Brief Intervention, and Referral to Treatment (SBIRT) to fill gaps in services and make referrals to specialty treatment when indicated [7]. SBIRT begins with universal screening using a validated screening measure to identify the level of SUD risk. For at-risk patients, screening is followed by a brief intervention tailored to the level of risk with the goal of increasing patient motivation or skills required to avoid substance use. When appropriate, brief intervention is followed by a referral to specialty care.

SBIRT has been found effective for tobacco use [8] and risky drinking [9]. However, the data on SBIRT for dependent alcohol use and for drug use are inconsistent [10]. Although 1 study found prepost reductions in alcohol and illicit drug use following SBIRT [11], a National Institute on Drug Abuse (NIDA)-funded randomized clinical trial of an SBIRT intervention—Assessing Screening Plus Brief Intervention’s Resulting Efficacy (ASPIRE) to Stop Drug Use—found no effects on any of the outcomes examined [12]. A separate study found positive effects for SBIRT in 3 countries, and a negative effect in the United States [13].

### Barriers to Screening, Brief Intervention, and Referral to Treatment

Barriers to delivering SBIRT in primary care may account for some of the negative outcomes regarding its efficacy. Barriers to screening and brief intervention for SUD include time constraints [14], fear of alienating the patients [15], and the challenge of working with patients with SUD and pain [16]. The barriers to referring patients for additional evaluation or specialty treatment include patient resistance [17,18], the stigma attached to treatment [19], and limited treatment resources [16]. There are additional challenges to implementing SBIRT for drug use as opposed to alcohol use. For example, the illegal nature of drug use raises concerns by patients and providers about privacy, and a brief intervention for drug use is more complicated than one for alcohol, as different drugs and patterns of use require different types of approaches to intervention [20]. Another challenge to making SBIRT work is ensuring, postvisit, that at-risk patients engage in appropriate self-management and adhere to treatment plans and referrals. A review of studies on dropout from SUD treatment programs revealed rates of dropout ranging from 21% to 43% for detoxification, 23% to 50% for outpatient, 17% to 57% for inpatient, and 32% to 68% for substitution (eg, methadone) treatment [21]. Among individuals who initially experience progress in treatment, relapse is common [22].

### A Stage-Based Mobile Intervention to Address Barriers to Screening, Brief Intervention, and Referral to Treatment

To integrate best practices and reduce barriers to SBIRT, a mobile-delivered substance use risk intervention (SURI) was developed. To address *patient barriers to SBIRT*, it was decided at the outset that SURI would be based on the transtheoretical model of behavior change (TTM), an empirically validated framework for matching interventions to readiness along a continuum of change. Behavior change involves progress through the following 5 stages: (1) precontemplation—not intending to make the behavior change in the next 6 months, (2) contemplation—intending to make the change in the next 6 months, (3) preparation—intending to make the change in the next 30 days, (4) action—made the change less than 6 months ago, and (5) maintenance—made the change more than 6 months ago. The TTM includes the following additional constructs central to change: (1) decisional balance—the pros and cons of changing [23], (2) self-efficacy—confidence to make and sustain the change in difficult situations [24], and (3) processes of change—10 cognitive, affective, and behavioral activities that facilitate progress through the stages [25,26]. More than 35 years of research on the TTM has identified particular principles and processes of change that work best in each stage to facilitate progress. The relationships between stage of change and these behavior change constructs provide an evidence-based framework for developing and delivering tailored feedback that is more likely to be remembered [27,28], considered personally relevant and credible [28-30], and to change behavior [28-30]. A meta-analysis found that health interventions tailored to stage produced significantly greater effects than those not tailored to stage [31]. A TTM approach can help facilitate progress through the stages of change for ending or reducing substance use, and for following through with treatment recommendations.

To address *system barriers to SBIRT*, it was also decided at the outset that SURI would rely on expert system technology, which could carry a significant part of the load in delivering SBIRT. Computer-tailored interventions (CTIs) based on the TTM have been found effective across a range of behaviors and populations, including smoking cessation [32], stress management [33], and depression management [34]. A recent trial of a TTM-based CTI and text messages for risky drinking found a strong effect on adherence to low-risk drinking limits (Levesque D et al, unpublished data, 2017). A CTI that shares information with the provider has the potential to also reduce barriers to communication, as individuals are more likely to disclose sensitive information to computers than to human clinicians [35,36]. The SURI prototype would include a risk assessment; a TTM-based CTI, text messages, and Web-based activities; and a clinical dashboard that summarizes the patient’s risk scores and stage of change data and provides stage-matched scripts to guide a brief in-person intervention session.

Existing Web-based and digital tools for SBIRT include provider-facing mobile apps that lead providers through an SBIRT screening [37] and, more recently, SBIRT screening tools embedded in the electronic health record (EHR) [38]. Although digital provider-facing screeners have the potential to increase provider confidence and reduce measurement error, they are time-consuming and do not address other barriers to SBIRT, such as discomfort in talking about substance use or patient resistance to change. A number of patient-facing Web-based programs and mobile apps have been developed for SUDs—most notably: (1) the *Alcohol Comprehensive Health Enhancement Support System* [39], a smartphone-based relapse prevention program that offers access to peer and professional support, reminders, education, and a Global Positioning System that identifies risky situations; (2) the *Therapeutic Education System* [40] *,* an interactive, Web-based psychosocial intervention with 65 interactive modules focusing on skills training; and (3) *Seva* [41], which combines the 2 programs above, and also includes a provider dashboard to help with patient monitoring. Although impressive and likely to have an impact on addictions, all 3 programs are designed for patients in recovery and are not appropriate for SBIRT.

The remainder of this manuscript describes the following steps taken to develop the stage-based SURI prototype:

1. Formative research—conducting a literature review and semistructured interviews with experts to provide guidance on the design specifications for the SURI tools
2. Intervention development—developing the intervention prototype based on the design specifications
3. Pilot testing—assessing the acceptability of the SURI tools in a pilot test involving providers and patients recruited from federally qualified health centers (FQHCs).

#### Formative Research

A literature review and semistructured interviews with expert consultants provided guidance on the development of the design specifications for the patient- and provider-facing SURI tools. A total of 5 experts brought expertise on SBIRT research, program development, and training; 2 experts—an SUD treatment agency chief executive officer and a health home team coordinator and peer counselor—brought expertise on the delivery of SUD specialty treatment; 1 expert was the director of a National Research Network and brought expertise on health information technology; and 1 expert brought expertise on mobile apps for substance use recovery.

Questions for the literature review and expert interviews included the following:

1. What are the barriers to delivering SBIRT in primary care?
2. How effective is SBIRT for drug use?
3. Are there any clues about “what works”?
4. *For screening*, which measures and which drugs to target?
5. *For brief intervention*, what content and what structure?
6. *For referral to treatment*, when to refer and what does referral entail?

Interviews, which lasted about 1.5 hours, were conducted by phone with 8 experts and in person with 1 expert. Examples of two key findings from the formative research and how they informed the design specifications for SURI development are as follows:

##### How to Select the Target Drug?

Findings from our review of 7 SBIRT outcome studies focusing on illicit drug use suggested that strategies for selecting the drug targeted in the SBIRT intervention may have an impact on outcomes. Studies that used a “flexible approach” or that allowed the patients to identify the target drug yielded negative or negligible effects [12,42,43], whereas studies that targeted a specific drug or class of drugs [44,45] or that relied at least in part on a validated risk assessment to identify the patient’s most problematic drug [13,46] yielded more positive effects. Although it is customary in SBIRT and other motivational enhancement interventions to invite patients to identify the behavior that concerns them most or that they are most ready to change, we may have a greater impact by focusing instead on the behavior causing the most harm.

For the SURI prototype, with expert guidance, we chose the World Health Organization’s (WHO) Alcohol, Smoking and Substance Involvement Screening Test (ASSIST) [47] to measure SUD risk and identify the patient’s most problematic drug. Up to 7 items are administered for each of 9 substances, with skip patterns for drugs that were never used or were not used in the past 3 months. For each substance, the ASSIST yields a risk score. Risk categories based on the scores—low (0-3), moderate (4-26), and high (27+)—have been extensively validated [47,48]. Studies have demonstrated the reliability and validity of the ASSIST administered by computer in a safety net population [49,50]—a method of administration used in a separate SBIRT intervention study that showed positive outcomes [46]. In the SURI prototype, the drug with the highest ASSIST score would be targeted in the intervention; to deal with ties, the program would use tie-breaker rules based on experts’ mean rankings of the drugs based on risks to health and well-being (opiates at the top and marijuana at the bottom).

##### What to do About the Highest-Risk Patients?

For individuals with a substance-specific ASSIST score in the high-risk range (27+), referral for further evaluation or specialty SUD treatment is indicated. Our review of SBIRT outcome studies revealed positive effects in 2 studies that excluded patients deemed high-risk based on the ASSIST or some other risk assessment [13,46], and negative or negligible effects in studies that included them [12,42-45]. However, among the studies that included them, protocols for treatment referral were not described at all [43,45] or were woefully inadequate, consisting only of providing the patients with a list of resources [12,42,44].

A TTM approach is ideally suited to increasing patient readiness to seek treatment, given the data on low treatment uptake [1] and high rates of dropout and drug relapse [21,22]. However, experts stressed that even as patients move forward in their readiness for change, they may not have the wherewithal to progress to action without additional help with understanding and weighing their treatment options, setting up the first appointment, and sticking to it. To address these needs, the SURI prototype would provide high-risk patients in the early stages of change for seeking treatment with information on different types of treatment and encourage patients and providers to discuss those options. For patients in the preparation stage, the program would encourage a “warm hand-off,” in which the patient and provider call the receiving agency to set up the first appointment. The SURI’s stage-matched text messages would include reminder messages in the days leading up to an appointment, which is an effective, low-cost method for increasing treatment attendance [51].

A second round of interviews with SBIRT and SUD experts provided specific ideas and language for the intervention content for each of the TTM modules. For example, for individuals in the precontemplation and contemplation stages, TTM interventions generally include a module designed to increase the “pros” or benefits of making the change—a concept that is consistent with motivational interviewing for SBIRT. SBIRT and SUD experts helped to identify the key pros of quitting a drug (eg, *so I can be a better parent* and *so I can feel more in control of my life*). Some pros were drug-specific and others were specific to the level of use.

#### Intervention Development

TTM-based CTIs tested in randomized trials generally include 3 CTI sessions delivered over 3 to 6 months [33,52-54] and text messages up to 6 months (Levesque D et al, unpublished data, 2017). However, the SURI prototype developed here included only the baseline CTI session and 30 days of text messages. SURI development required documenting measures and decision rules for scoring the measures and delivering tailored feedback, writing intervention content, programming the decision rules, developing the look and feel, and testing and debugging. SURI prototype components include the following:

1. *Patient-facing computer-tailored intervention*: The SURI CTI session was accessible via an Internet-enabled smartphone, computer, or tablet computer, and could be completed at home or in the primary care clinic. The session’s general session flow, designed to address all components of SBIRT—screening, brief intervention, and referral to treatment–was as follows: 
  - First, assess SUD risk using the ASSIST [47] and present a chart showing the patient’s level of risk (none, low, moderate, or high) for health and other problems associated with each class of drugs assessed.
  - Second, assign the patient to an intervention track based on the most problematic drug and level of risk (moderate vs high). Screen out low-risk patients.
  - Third, inform the patient about his or her most problematic drug, and provide education on the specific health risks associated with that drug following procedures outlined in the WHO’s ASSIST manual [55].
  - Fourth, assess readiness to quit the most problematic drug and deliver a brief stage-matched intervention representing 3 key processes and principles of change for that stage, encourage the patients to set at least one stage-matched goal from the list provided, and assist in making a simple plan for goal implementation [56].
  - Finally, assess readiness to seek treatment if the ASSIST score is ≥27 for the most problematic drug, and deliver a brief stage-matched intervention that includes information about treatment options.
2. *Stage-matched text messages*: Short message service messages for 30 days were tailored to the patient’s most problematic drug, level of risk, and stage of change for quitting and for seeking treatment, if indicated. Text messages were delivered every 1 to 3 days, depending on the stage of change. One text message each week contained a link to an interactive Personal Activity Center (PAC) activity. Sample text messages for a high-risk stimulant user in the contemplation stage for quitting included the following: (1) *Is the thought of having cravings keeping you from cutting back on your stimulant use? Learn how to deal with cravings at [link to PAC activity],* and (2) *How much do you know about stimulants? Check out drugabuse.gov. Once you learn more, you can decide if you want to cut back or stop using them.*
3. *PAC activities:* This included brief interactive activities, accessible via text messages and email, which focused on key topics (eg, dealing with cravings and working on negative thinking) for making positive changes in substance use behavior.
4. *Clinical dashboard:* This included a provider-facing tool that displayed an overview of the patient’s CTI session data and provided scripts for a brief in-person intervention session matched to the patient’s stage of change for quitting the most problematic drug and for seeking treatment, if indicated. In the SURI prototype, the dashboard was accessible via a link from the clinic EHR. Providers entered the patient’s name and date of birth to retrieve the patient’s dashboard.
5. *Printable dashboard summary*: This included a portable document format (PDF) summary of the dashboard content, along with a list of local referral resources.
6. *Patient report:* This included a PDF of all the feedback the patient received during the SURI session, along with additional questions and resources.

Program screenshots are provided in Multimedia Appendix 1. The Flesch-Kincaid reading level for patient-facing content is 5.0. All decision rules, content, and the final working prototype were reviewed by experts, revised, and rereviewed. No subjects were recruited to provide feedback during the formative research or intervention development phases of the study. Given the funding source for this study, research involving the collection of data from more than 9 respondents required clearance by the US Office of Management and Budget, and it was not practical to seek clearance in the 6-month project period. All 9 subjects, which included providers, were reserved for the pilot test described below.

#### Pilot Test

The remainder of this report describes a pilot test conducted to gather preliminary data on the acceptability of the mobile-delivered SURI program and clinical dashboard on a sample of 9 providers and patients. Acceptability would be established if providers and patients perceived the mobile tools as acceptable and useful—as evidenced by overall mean ratings of at least 4.0 on 5-point acceptability measures.

Providers and patients provided written informed consent for the pilot test. The Pro-Change Institutional Review Board approved the study.

## Methods

### Participants

#### Providers

As the sample size was limited to 9 subjects, including providers, it was not possible to implement universal SURI screening as a method for identifying patients at risk for SUD. Instead, 4 providers—1 from a FQHC in Georgia and 3 from a FQHC in Rhode Island—were recruited to participate in the study, and each was to recruit 1 or 2 patients. The 4 providers included a physician, 2 physician’s assistants, and 1 family nurse practitioner. They had an average age of 40.5 years (standard deviation [SD] 6.8); 3 were female, 3 were white non-Hispanic, and 1 was Hispanic. The Georgia provider was unsuccessful in recruiting patients, and attributed her difficulty to her recent arrival at the FQHC; she had not yet had the opportunity to build the necessary rapport with patients. The Georgia provider participated in other study activities that did not involve patients. The 3 Rhode Island providers recruited 5 patients to yield a total study sample of n=9. The Rhode Island FQHC is a federally qualified, Joint Commission-accredited Level 3 Patient-Centered Medical Home, which offers a full range of clinical services to over 13,000 culturally diverse patients per year. Providers were offered US $450 for participating in 2 interviews, completing a brief training on the dashboard, recruiting patients, and delivering an in-person dashboard-guided session to the patients they recruited.

#### Patients

Providers reached out to patients by phone or during a scheduled office visit to describe the study and invite them to participate. Those interested called the study team using a toll-free number provided. The 5 patients had an average age of 41.8 years (SD 13.6), 4 were male and 1 was male-to-female transgender, 3 were white non-Hispanic and 2 were Hispanic, and all were unemployed. Patients received a total of US $110 for participating in 4 interviews, a Web-based SURI session, and an in-person dashboard-guided session with their provider.

### Procedure

#### Providers

All 4 providers took part in an initial 30-min interview asking about barriers and facilitators to SBIRT, current clinic policies, and personal opinions and practices regarding SBIRT. They also participated in a 30-min webinar training. The 3 Rhode Island providers recruited 5 patients and completed a dashboard-guided SBIRT session with 4 of them, as 1 patient dropped out before initiating his SURI CTI session. The 3 providers also participated in a final interview that included a 6-item acceptability measure containing questions adapted from NCI’s Education Materials Review Form [57] and a 5-item measure [58] that has been used to evaluate other tailored and stage-matched intervention materials [59,60]. Response options for the acceptability measure ranged from 1 (strongly disagree) to 5 (strongly agree). The criterion for establishing acceptability in the pilot test was an overall rating of 4.0 or higher across items. Providers also answered follow-up questions on what they liked most and least about the dashboard, and about EHR and clinical flow integration and training needs. NVivo software (QSR International) was used for the qualitative analysis of the interview content using node reports to identify themes and patterns in provider responses.

#### Patients

Before completing the SURI CTI session, the 5 patients met with a member of the project team at the clinic or by phone to answer questions on demographics and prior experience with SBIRT in primary care, and to give feedback on the intervention title, logo, and introduction screen. One patient chose to discontinue his involvement in the study before completing the interview or starting the SURI CTI session. He said that he felt the interview questions were too personal. Data from the SURI session show that 3 of the 4 patients who completed the session were polysubstance users. For 2 patients, the most problematic drug was opioids (ASSIST scores of 21 and 30), and for 2, it was cocaine (ASSIST scores of 27 and 29). Furthermore, 2 patients were in the contemplation stage, 1 was in action, and 1 in maintenance.

After completing their SURI CTI session, patients participated in an in-person SBIRT session with their provider and accessed other program components during the next 30 days. They also completed 3 acceptability surveys following the same format as the provider surveys. The first survey, administered after the SURI CTI and in-person SBIRT session, included 10 questions assessing the acceptability of the SURI CTI session. The second and third surveys, administered 2 and 4 weeks later, included 8 questions assessing the acceptability of the text messages. Patients were also asked to report what they liked most and least about the various program components and what they found most helpful about the one-on-one meeting with their provider.

## Results

### Providers

Providers delivered dashboard-guided intervention sessions to all pilot test participants who completed a SURI CTI session. All elements of the dashboard functioned as intended.

#### Acceptability of the Clinical Dashboard

On the basis of self-report, providers spent an average of 11.2 min (SD 9.5) discussing the dashboard with their patients. Table 1 shows providers’ mean ratings on the 6 dashboard acceptability dimensions. The overall mean rating across items was 4.4 (SD 0.4), which exceeded the study benchmark of 4.0 for acceptability. When asked what they *liked most* about the dashboard, common themes emerged:

- All used the ASSIST scores and agreed with the program’s decision regarding the patient’s most problematic drug.
- All mentioned that the dashboard taught or gave them something new to discuss with their patient.
- Two providers mentioned that their patient was thinking about the process of quitting differently. One provider said that the CTI session had planted a seed and the text messages were helping it to germinate.
- Two providers used the dashboard as a visual aid to facilitate communication with their patient. They said it allowed the patient and the provider to start on a common ground and work toward a mutual goal.
- All stated that the dashboard gave them a clear, concise, attractive visual representation of the patient’s data, which allowed for a structured conversation focusing on the most problematic drug.

When asked what they *liked least*, 1 provider commented that the dashboard did not accurately capture his patient’s stage of change. Upon further discussion, we realized that the patient had placed herself in maintenance because she had quit using cocaine on weekdays (though still used on weekends).

#### Clinical Flow Integration

The dashboard was easily integrated into clinical flow at the FQHC during the pilot test, particularly for the 3 patients who had completed the SURI CTI session at home. Providers supported the idea of patients completing the CTI session at home. However, they also felt that the SURI CTI session could be administered in the waiting room on a mobile device that could be carried into the exam room, if needed. They stated that incorporating other staff to facilitate the use of the tool would be imperative.

#### Electronic Health Record Integration

Providers stated that the need to search for the patient’s dashboard would be a major barrier to implementation. When prompted to identify what dashboard-EHR integration would ideally look like, providers recommended the following: (1) alerts in the EHR when a new dashboard becomes available, or when an existing dashboard is updated; (2) once in a patient’s EHR, the ability to gain access to that patient’s dashboard with a single click; (3) the ability to set and track patient goals or action steps in the dashboard; and (4) the ability to save the following data to the EHR: ASSIST drug risk scores, stage of change, any action steps, and the fact that a brief intervention focusing on substance use was conducted. These recommendations aligned with those of SBIRT experts who reviewed the intervention.

#### Training

To use the dashboard effectively and comfortably, providers agreed that a training session is needed and suggested a 30-min session like the one they had received.

**Table 1:** Acceptability of the dashboard among providers.

Acceptability dimension (n=3)	Mean rating (SD^a^)
The program was easy to use	4.3 (0.6)
The data were easy to understand	4.7 (0.6)
I like the way the program looked	4.3 (0.6)
The program could help my patient make some positive changes	3.7 (1.5)
The program can give me helpful information about my patient	5.0 (0.0)
I would be willing to use this program again	4.3 (0.6)

^a^SD: standard deviation.

**Table 2:** Acceptability of the substance use risk intervention computer-tailored intervention session among patients.

Acceptability dimension (n=4)	Mean rating (SD^a^)
The program was easy to use	5.0 (0.0)
The questions were easy to understand	4.8 (0.5)
The personal feedback was easy to understand	4.5 (0.6)
I like the way the program looked	4.3 (0.5)
I felt the program respected my thoughts and point of view	4.0 (0.0)
The program gave me new things to think about	4.5 (1.0)
The program could help me make some positive changes	4.5 (0.6)
The program can give my provider helpful information about me	4.5 (0.6)
I would be willing to use this program again	4.5 (0.6)
I know someone else who could benefit from this program	4.3 (1.0)

^a^SD: standard deviation.

### Patients

On the basis of the self-report and program utilization data, all 4 pilot test participants accessed all program components (SURI CTI session, text messages, and PAC activities) during the first 2 weeks of the intervention period; 3 of 4 participants accessed all available program components (text messages and PAC activities) during the final 2 weeks. All patient-facing SURI components functioned as intended.

#### Acceptability of Substance Use Risk Intervention Computer-Tailored Intervention Session

Table 2 shows the 10 SURI acceptability dimensions and their mean ratings among patients. The overall mean rating was 4.5 (SD 0.3), which exceeded the benchmark for acceptability. These ratings are impressive, particularly in light of the fact that there were no opportunities to elicit patient feedback on the intervention during development. All 4 patients responded to the question asking what they *liked most* about their Web-based session. For example:

I could do it at my own time. I could think about my answers and there is no person giving you feedback right away. There was no judgment.

Furthermore, 2 patients responded to the question asking what they *liked least*:

It was little bit long. Not too many questions, but it just seemed to take a while.

#### Time to Complete Substance Use Risk Intervention Computer-Tailored Intervention Session

We had expected SURI CTI sessions, such as sessions for other TTM-based CTI programs, to take about 20 min to complete. However, Google Analytics showed that sessions took an average of 35.6 min (SD 14.1).

#### Helpfulness of In-Person Session

A total of 3 patients responded to the question regarding what they found most helpful about their in-person session. For example, one patient stated the following:

Well that she did review the online questionnaire I did, and the suggestions she had really hit home: I usually don’t talk to people and she suggested that I need to talk to others about it. In a way it made me a little worried but towards the end I was more comfortable.

#### Acceptability of Text Messages

All participants completed the 2-week assessment examining the acceptability of the text messages, and 3 of the 4 participants completed the 4-week assessment. Patients’ 2- and 4-week acceptability ratings were nearly identical, so their ratings were averaged. Table 3 shows mean ratings for the 8 acceptability dimensions among patients. The lowest mean rating was for the item, *The text messages were easy to understand* (3.6), and highest was for the item, *Reading the text messages was worth the time it took* (4.8). The overall mean rating was 4.0 (SD 0.4), which met the benchmark for acceptability. When asked what they liked most about the text messages, 1 participant responded:

That it was a reminder that I am doing this. I tend to forget and it’s nice to have this reminder so I don’t forget. And it helps me to plan my days and avoid the temptations that I can.

When asked what they liked least, 1 participant responded:

I have a disability and don’t always understand the questions in the text messages. It’s hard to get the point of it. I can’t ask a phone to explain what you mean by a text.

On the basis of these and other responses, we will pursue the following kinds of improvements to text messages in future work: (1) conduct focus group to help ensure that text messages are understood and interpreted as intended, (2) allow patients to specify the time of day that they receive text messages, (3) allow 2-way texting, (4) provide an email option, and (5) add more links to resources—for example, information and sources of help.

**Table 3:** Acceptability of the substance use risk intervention text messages.

Acceptability dimension (n=4)	Mean rating (SD^a^)
The text messages were easy to understand	3.6 (1.8)
The text messages reinforced things I learned in the online session	4.1 (0.9)
The text messages were supportive	3.8 (1.0)
The text messages gave me new things to think about	4.5 (0.6)
The text messages could help me make some positive changes	3.6 (1.8)
Reading the text messages was worth the time it took	4.8 (0.5)
The text messages arrived at times when it was good for me to receive them	4.0 (1.2)
I know someone else who could benefit from messages like these	3.7 (1.5)

^a^SD: standard deviation.

#### A Follow-Up Message From a Participant

The patient who did not participate in the 4-week survey contacted the study’s project manager approximately 4 months later and gave his consent for us to share his message:

I found your number in an email and I wanted to let you know that I have been sober for 7 weeks. It’s the longest I’ve been off opioids besides the year I was in prison. It’s amazing. I’m sorry I didn’t complete the final activity. I wanted to know if I could still complete it. I met with [provider] today and she didn’t think I could do it. But I did it. I can only move up from here.

Although it is impossible to attribute this patient’s positive changes to his involvement in the pilot test, it is interesting to note that he chose to share his success with both his provider and a member of the study team.

## Discussion

### Principal Findings

To help address the barriers to SBIRT, the SURI tools were designed to: (1) reduce provider time and burden, (2) facilitate patient-provider communication, (3) facilitate evidence- and risk-based decision making that accommodates a range of drugs and patterns of use, (4) increase provider adherence to best practices, (5) increase provider comfort and confidence, and (6) facilitate patient readiness to quit their most problematic drug and, if indicated, to follow through with treatment recommendations. All program components functioned as intended, and SURI program acceptability ratings from both providers and patients met or exceeded the criteria for establishing acceptability. Patient ratings are especially impressive, in light of the fact that there were no opportunities to elicit patient feedback on the intervention during development.

It is noteworthy that the provider who was unsuccessful in recruiting patients for the pilot test attributed the problem to her lack of rapport with patients, as she was relatively new to her clinic. And the patient who chose to discontinue his involvement during the first interview attributed his decision to discomfort with the study questions. No doubt, discussing substance use—and especially drug use—can be uncomfortable for both providers and patients. Although this discomfort may serve as a barrier to using the SURI tools, it is also possible that relying on SURI to introduce and deliver universal screening and a brief intervention via mobile tools, as a part of routine care, can reduce stigma and open a channel for patient-provider communication focused on patient health and well-being. A SURI demonstration project that allows universal screening is required to assess the program’s acceptance and uptake among providers and patients under typical clinic conditions.

#### Substance Use Risk Intervention Program Enhancements

Only a prototype of the SURI program was developed here. Steps to complete intervention development would include the following: (1) revising intervention content and procedures based on the current findings and recommendations; (2) developing content for the second and third SURI CTI sessions; (3) writing an additional 5 months of text messages and additional PAC activities; and (4) evaluating all content for reading level (ensuring grade 5) and cultural sensitivity and revising as necessary. As Hispanic Americans comprise about 17% of the US population [61] and 34% of FQHC patients [62], translation of all patient-facing components into Spanish would be necessary to increase SURI program accessibility and disseminability.

The 2 follow-up CTI sessions would be similar in flow and structure to the baseline session described above. However, follow-up sessions would also inform participants on how they have changed on the following 4 key dimensions: (1) the drug identified as most problematic, (2) level of risk for that drug, (3) stage of change for quitting that drug, and (4) stage of change for seeking treatment, if indicated. Guidance delivered in the CTI sessions and text messages, and content in the PAC activities, would be matched to the updated data regarding the most problematic drug, risk level, and stage of change. These updated data would also be shared with the provider via the dashboard and trigger updated stage-matched scripts for new one-on-one sessions. The 3 SURI CTI sessions could be delivered over 3 or 6 months.

Moving forward, a more participatory approach will be required to refine and enhance the intervention based on the pilot test findings, and to develop the remainder of the intervention package. Patient focus groups in future research will review the operational definition of “quit,” and will review all other intervention content to ensure it is interpreted as intended. Usability tests using a variety of devices (smartphone, tablet computer, and computer) will ensure that all program components are easily navigable, and will examine how long the SURI CTI session takes to complete and how participants spend their time. In addition, interviews with patients, experts, and providers can help to identify an acceptable session length for clinic administration (we suspect 20 min), and whether to scale back on intervention content to reduce session length—for example, by reserving some SURI content for PAC activities or follow-up SURI sessions.

In the research described above, a final round of interviews was conducted with 7 experts to outline plans for integrating the mobile tools with one or more EHR products and within clinical practice. Experts agreed that SURI integration with the EHR was essential, and their vision of what that should look like matched that of providers as described above. Experts also identified the following program features and functions deemed necessary to maximize the program’s usability, impact, and likelihood of clinic-wide adoption in a large randomized trial and, eventually, under real-world conditions:

1. Allow the provider to *override SURI decision rules* regarding the most problematic drug and need for treatment. For example, a provider may want SURI to focus on a different drug that poses a great risk for the patient, given a cooccurring medical problem, or want a patient who falls below the ASSIST treatment cut point to seek treatment. We will also allow the provider to request that the patient’s stage of change be reevaluated by the SURI program. This request will trigger a text message linking the patient to a brief stage assessment.
2. Allow the provider to *select specific action steps* that could be communicated to SURI (eg, cut back on drug X by Y amount).
3. Program SURI to *monitor patients’ progress on action steps* and *communicate that progress back to the clinic*. Adhering to patient privacy rules, SURI would not communicate with outside treatment providers or care organizations. Rather, it would rely on text messaging to elicit patient reports on their progress on action steps, and share patient responses with a designated patient care manager at the clinic, who could then take any appropriate steps required.

Features 2 and 3 above deepen SURI’s integration with clinical practice and have the potential to increase SBIRT’s impact, particularly among the highest-risk patients requiring further SUD evaluation or treatment.

### Limitations

The pilot test was small and conducted under ideal conditions. It would be unrealistic to expect that level of enthusiasm and adherence when the intervention is rolled out in a large clinical trial to assess its efficacy or in the real world. Future research involving clinic-wide implementation would require a “make it happen” approach [63] to implementation. Making it happen would need to involve several best practices from implementation science, such as: (1) ensuring buy-in from leadership [64]; (2) assembling members of an implementation team [64] at each site to define site goals for screening and SBIRT delivery (percentage of patients screened and percentage of eligible patients who receive an in-person dashboard-guided session); (3) assembling members of an implementation team [64] at each site to outline practicable procedures that will support universal screening, timely in-person SBIRT sessions, and appropriate follow-up from a care manager; (4) identifying and training a champion at each site who can serve as a role model for the implementation [64]; (5) providing training to staff and providers; and (6) using plan-do-study-act cycles [65,66] once the implementation has started—in this case, using scores on key metrics to guide improvements in implementation procedures and outcomes over time, in an iterative fashion. Key metrics could include provider acceptability ratings; number of SBIRT screenings completed each week; number of moderate- and high-risk patients identified via screening; and among the patients identified, the percentage receiving an in-person SBIRT session. Low ratings at a given site could trigger an exploration of problems and barriers at the site, as well as efforts to find solutions.

### Conclusions

This study represents a large step forward in the development of mobile tools that have the potential to reduce major barriers to SBIRT. The SURI program’s particular combination of features, along with future enhancements and efforts to integrate SURI into clinical flow and the EHR, will be uniquely designed to help providers deliver all 3 elements of SBIRT—screening, brief intervention, and referral to treatment—with efficiency and adherence to evidence-based practices—within a busy primary care setting.

## Appendix

Multimedia Appendix 1 SURI (Substance Use Risk Intervention) program screenshots.

## Abbreviations

ASSIST: Alcohol, Smoking and Substance Involvement Screening Test
ASPIRE: Assessing Screening Plus Brief Intervention’s Resulting Efficacy
CTIs: computer-tailored interventions
EHR: electronic health record
FQHC: federally qualified health center
NIDA: National Institute on Drug Abuse
PAC: Personal Activity Center
PDF: portable document format
SBIRT: Screening, Brief Intervention, and Referral to Treatment
SD: standard deviation
SUD: substance use disorder
SURI: substance use risk intervention
TTM: transtheoretical model of behavior change
WHO: World Health Organization
