# Supplementary material for: Stage-Based Mobile Intervention for Substance Use Disorders in Primary Care: Development and Test of Acceptability
Source: JMIR Med Inform. 2018 Jan 2;6(1):e1. doi: 10.2196/medinform.7355 (PMC5770579; doi:10.2196/medinform.7355)
Supplement: Multimedia Appendix 1 [file medinform_v6i1e1_app1.pdf]

Substance Use Risk Intervention Screenshots

Transition from screening to brief intervention

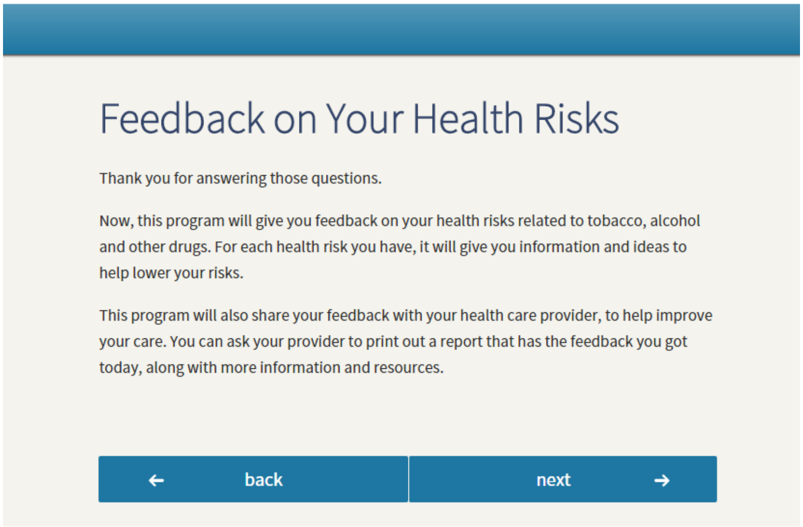

©2017, Pro-Change Behavior Systems, Inc.

Feedback on substance use-related risk, based on the World Health Organization's (WHO) Alcohol, Smoking and Substance Involvement Screening Test (ASSIST)

Drug Use

Now, here is your **risk** for health or other problems related to other drugs, based on your pattern of use.

Drug	Your risk for health and other problems
Cannabis (marijuana, pot, grass, weed, hash, etc.)	Moderate
Cocaine (coke, crack, etc.)	Low
Amphetamine-type stimulants (speed, crystal meth, ice, Dexedrine, Adderall, diet pills, etc.)	High
Inhalants (nitrous, glue, petrol, paint thinner, etc.)	None
Sedatives or sleeping pills (Valium, Serepax, Ativan, Xanax, Librium, Rohypnol, GHB, Ambien, Lunesta, etc.)	Moderate
Hallucinogens (LSD, acid, mushrooms, PCP, Special K, ecstasy, etc.)	Low
Opioids (heroin, opium, fentanyl, oxycodone [OxyContin, Percocet], hydrocodone [Vicodin], methadone, buprenorphine, etc.)	Past
Other (Kedimine)	Low

©2017, Pro-Change Behavior Systems, Inc.

Feedback on most problematic drug, including health risks taken from the WHO's guidelines for SBIRT intervention

Stimulants

(speed, crystal meth, ice, Dexedrine, Adderall, diet pills, etc.)

Based on your pattern of drug use, **amphetamine-type stimulants** are causing the most serious risks to your health and well-being. There is a good chance that you are addicted to stimulants.

Here are some of the risks associated with stimulant use:

More serious risk

- Problems sleeping, loss of appetite and weight loss, fluid loss, and trouble fighting infections
- Jaw clenching, headaches, and muscle pain
- Mood swings—anxiety, depression, mania, and panic
- Tremors, irregular heartbeat, and shortness of breath
- Trouble focusing and remembering things
- Paranoia, aggressive and violent behavior
- Psychosis after repeated use of high doses
- Lasting damage to brain cells
- Liver damage, bleeding in the brain, and sudden death from acute heart conditions

©2017, Pro-Change Behavior Systems, Inc.

Feedback on stage of change

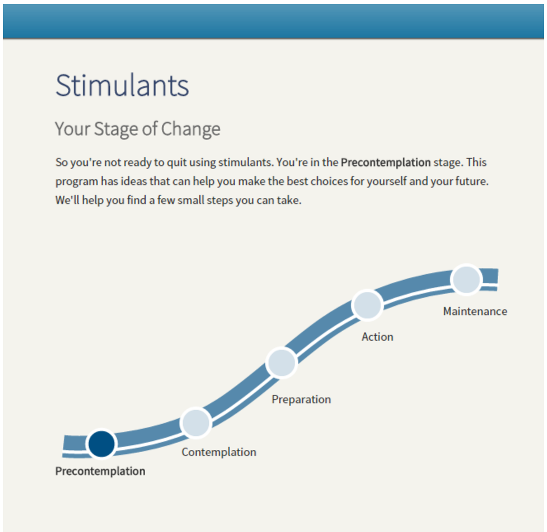

©2017, Pro-Change Behavior Systems, Inc.

Stage-matched guidance designed to facilitate progress to the next stage of change

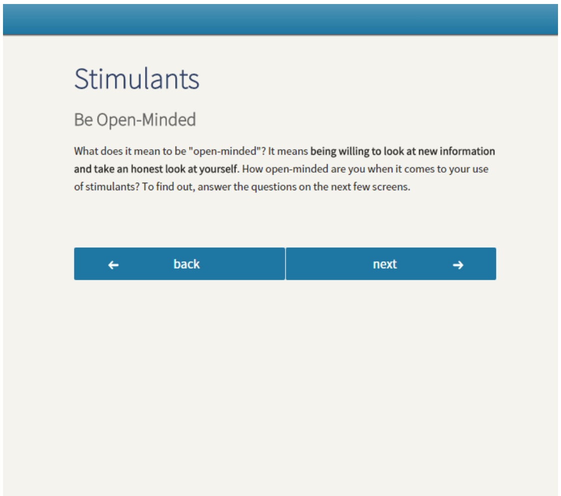

©2017, Pro-Change Behavior Systems, Inc.

Introduction to help seeking

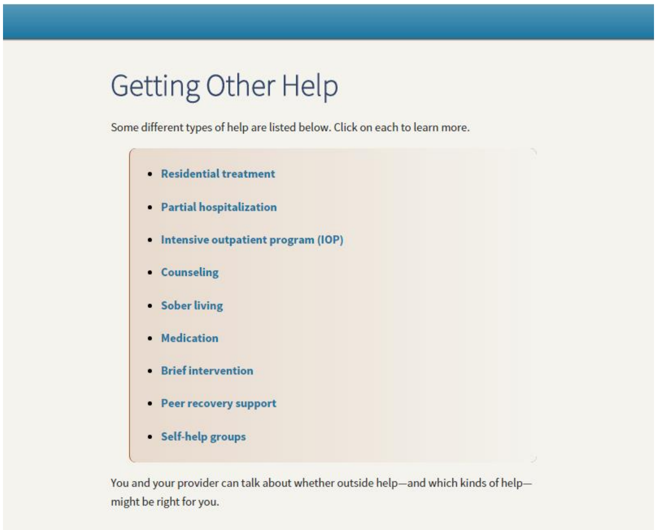

©2017, Pro-Change Behavior Systems, Inc.

Help seeking stage of change assessment

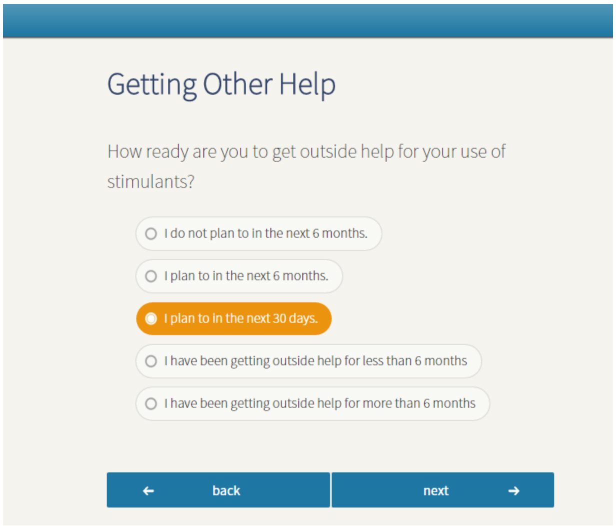

©2017, Pro-Change Behavior Systems, Inc.

Stage-matched text message

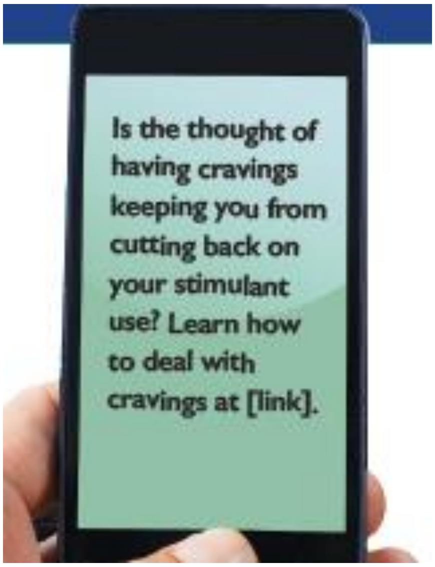

©2017, Pro-Change Behavior Systems, Inc.
pro-change BEHAVIOR SYSTEMS, INC.

Personal Activity Center (PAC) activity accessible via text message

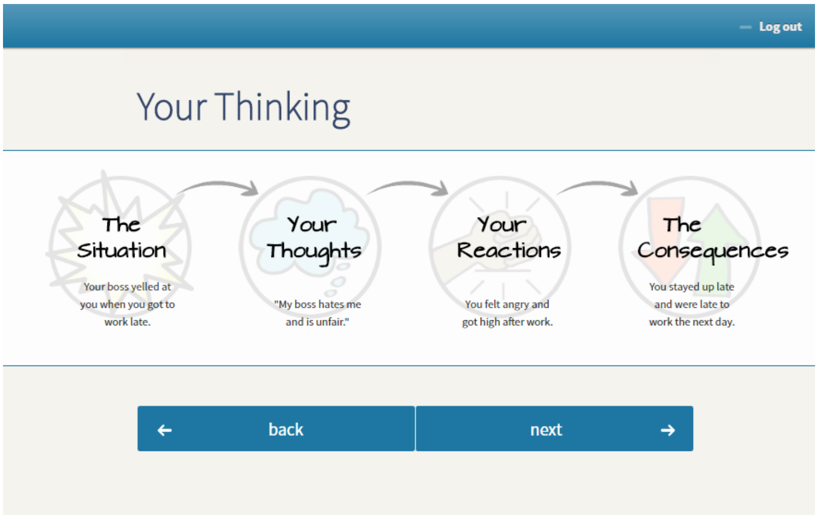

©2017, Pro-Change Behavior Systems, Inc.
pro-change BEHAVIOR SYSTEMS, INC.

Clinical Dashboard for providers

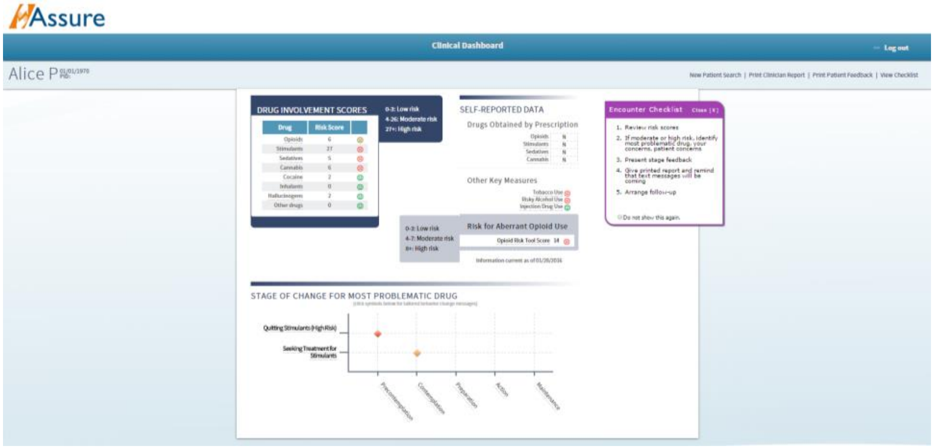

©2017, Pro-Change Behavior Systems, Inc.

Clinical Dashboard stage-matched script

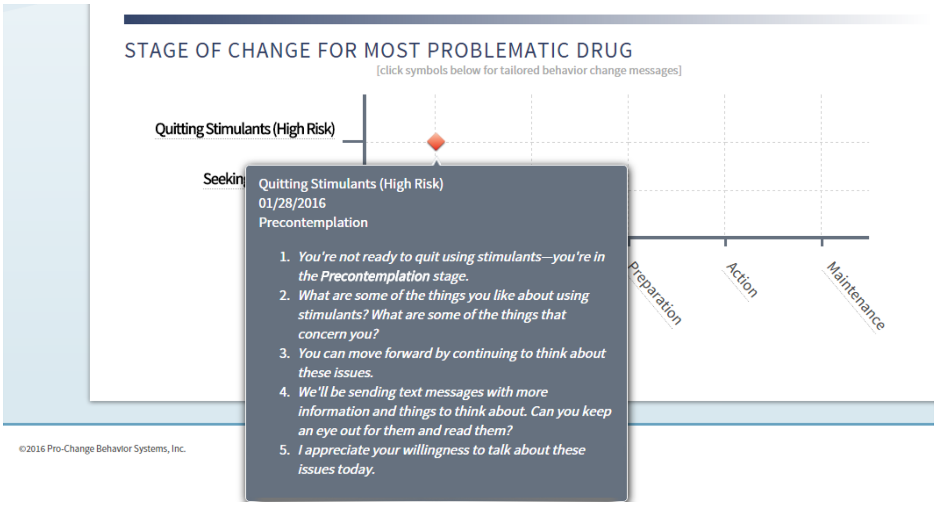

©2017, Pro-Change Behavior Systems, Inc.
